# Mucin Secretion Induced by Titanium Dioxide Nanoparticles

**DOI:** 10.1371/journal.pone.0016198

**Published:** 2011-01-19

**Authors:** Eric Y. T. Chen, Maria Garnica, Yung-Chen Wang, Chi-Shuo Chen, Wei-Chun Chin

**Affiliations:** Bioengineering, University of California Merced, Merced, California, United States of America; University of Pennsylvania, United States of America

## Abstract

Nanoparticle (NP) exposure has been closely associated with the exacerbation and pathophysiology of many respiratory diseases such as Chronic Obstructive Pulmonary Disease (COPD) and asthma. Mucus hypersecretion and accumulation in the airway are major clinical manifestations commonly found in these diseases. Among a broad spectrum of NPs, titanium dioxide (TiO_2_), one of the PM10 components, is widely utilized in the nanoindustry for manufacturing and processing of various commercial products. Although TiO_2_ NPs have been shown to induce cellular nanotoxicity and emphysema-like symptoms, whether TiO_2_ NPs can directly induce mucus secretion from airway cells is currently unknown. Herein, we showed that TiO_2_ NPs (<75 nm) can directly stimulate mucin secretion from human bronchial ChaGo-K1 epithelial cells via a Ca^2+^ signaling mediated pathway. The amount of mucin secreted was quantified with enzyme-linked lectin assay (ELLA). The corresponding changes in cytosolic Ca^2+^ concentration were monitored with Rhod-2, a fluorescent Ca^2+^ dye. We found that TiO_2_ NP-evoked mucin secretion was a function of increasing intracellular Ca^2+^ concentration resulting from an extracellular Ca^2+^ influx via membrane Ca^2+^ channels and cytosolic ER Ca^2+^ release. The calcium-induced calcium release (CICR) mechanism played a major role in further amplifying the intracellular Ca^2+^ signal and in sustaining a cytosolic Ca^2+^ increase. This study provides a potential mechanistic link between airborne NPs and the pathoetiology of pulmonary diseases involving mucus hypersecretion.

## Introduction

Many published reports have demonstrated the association between NP exposure and pulmonary morbidity and mortality [1], [2], [3]. The adverse effects induced by NPs seem to exacerbate clinical symptoms of pre-existing respiratory illnesses such as asthma, COPD and Cystic Fibrosis (CF) [1], [2], [3], [4], [5], [6]. During NP exposure, individuals with respiratory diseases showed more incidences of bronchoconstriction, medication use, bronchial hyperreactivity and lung fibrosis [2], [7]. TiO_2_ NPs are widely used in the nanotechnology industry due to their vast array of applications that range from household commodities, such as components of paints and carpets, to personal products that include cosmetics, textiles, sunscreens and foods [8], [9]. TiO_2_ is also one of the PM10 components commonly found in industries or manufacturing plants involved in processing mineral ore rutile [10]. It has been reported that >50% of TiO_2_ NP exposed workers had respiratory symptoms accompanied by reduction in pulmonary function [10], [11]. Other reports have also indicated that inhalation of TiO_2_ NPs can induce pulmonary inflammatory responses (characterized by neutrophil recruitment), epithelial cell death and increased permeability [2], [9]. Furthermore, TiO_2_ NPs have been shown to play a role in inducing epithelial fibroproliferative changes, stimulating goblet cell hyperplasia and in instigating emphysema-like (such as alveolar enlargement) damages in the lungs [2], [10], [12]. Overall, nanotoxicity induced by TiO_2_ NP exposure in both the occupational and ambient environment presents a significant and realistic health concern.

The harmful effects of NPs on the respiratory system not only encompass cellular apoptosis/necrosis, but also mucus hyperproduction which is closely associated with the pathogenesis of pulmonary diseases that include asthma, COPD and CF [2], [10], [13]. In these chronic pulmonary diseases, mucus hypersecretion and accumulation may lead to recurrent episodes of chronic bacterial infections, limited airflow and chronic inflammatory responses [2], [14], [15]. However, whether TiO_2_ NPs can directly induce mucin secretion has not been resolved.

Airway mucus plays a vital role in the constant clearance of inhaled pathogens and particulates. Mucus is a large, highly glycosylated protein consisting of an array of mucin peptides (apomucin) [14]. With their oligosaccharide sidegroups, such as sialic acid, sulfate, and carboxyl (COO^−^), mucins are usually polyanionic in nature [16]. Mucin secretion is closely regulated by cytosolic Ca^2+^ concentrations ([Ca^2+^]_C_) in various epithelial cells [17]. A rise in [Ca^2+^]_C_ is crucial for initiating a cascade of down stream events including the mobilization of granule-bound Ca^2+^, docking of the secretory granules, fusion of the plasma-granule membrane and the formation of secretory pores, therefore leading to the exocytosis of the mucin granules [18].

Agonist-induced opening of various Ca^2+^ channels expressed on the cell membrane allows the influx of extracellular Ca^2+^, which may serve as the external Ca^2+^ source [19]. The initial upsurge in the [Ca^2+^]_C_ is usually relayed by triggering a secondary wave of Ca^2+^ propagation from internal stores, such as the ER [19], [20], [21], [22]. Ryanodine receptors (RYRs) on the ER have multiple allosteric Ca^2+^ binding sites responsible for triggering Ca^2+^- induced Ca^2+^ release (CICR) into the cytosol [19], [20], [21], [22]. The resultant increase in [Ca^2+^]_C_ could activate other cytosolic proteins and modulate secretion of mucin, hormones or various neurotransmitters [17], [23], [24].

NPs have been shown to disturb cellular functions by elevating intracellular Ca^2+^ levels [25], [26], [27], [28]. For example, ultrafine carbon black NPs can elicit Ca^2+^-dependent secretion through the activation of L-type voltage-gated Ca^2+^ channels [25], [26], [28]. However, little is known regarding the intricate calcium signaling pathway regulating the exocytotic events of secretory products. In this study, we aim to investigate the mechanism through which TiO_2_ NPs induce mucin secretion via a Ca^2+^ signaling mediated pathway.

## Materials and Methods

### 1. Culture of ChaGo-K1 cells

The human airway bronchial epithelial cell line ChaGo-K1, obtained from American Type Culture Collection (ATCC, Manassas, VA, USA), was used because it expresses MUC proteins and secretes mucin [29]. Cells were cultured in 15 cm cell culture plates (VWR, CA, USA) in RPMI 1640 medium (Invitrogen, CA, USA) supplemented with L-glutamine, 1% penicillin/streptomycin and 10% heat inactivated fetal bovine serum (FBS). Cultures were incubated in a humidified incubator at 37°C/5% CO_2_. Cell counts were performed using trypan blue (Sigma-Aldrich, MO, USA) exclusion and a Bright-Line haemocytometer.

### 2. Nanoparticles and characterization

A mixture of anatase and rutile forms of ultrafine titanium (IV) dioxide (<75 nm) (Sigma-Aldrich, MO, USA) was used in this study because this form has been shown to result in more severe cellular injuries [30], [31]. The TiO_2_ NPs have a surface area of 36 m^2^/g and the dispersion conductivity is 1040 µS/cm (information from Sigma). All NP samples were sonicated before usage. The concentrations used were 1 mg/ml, 0.75 mg/ml, 0.5 mg/ml, 0.25 mg/ml, 0.1 mg/ml, and 0.05 mg/ml. The range of concentrations used was consistent with the concentrations of TiO_2_ NPs found in previous reports [30]. The TiO_2_ NPs were reconstituted with Hanks' solution (Invitrogen, CA, USA) before being tested. The size of NPs was independently confirmed using homodyne dynamics laser scattering (DLS) as described in previous studies [32], [33].

### 3. Cell preparation

Cells were seeded at 2×10^5^ cells per well in a 24-well plate, and incubated for 24 hrs in RPMI 1640 supplemented with 10% FBS. Following 24 hr incubation, the RPMI medium was removed from the cells and the culture was rinsed with Hanks' solution twice before use.

### 4. Measurements of cytosolic Ca^2+^ concentrations induced by TiO_2_ exposure

The cells were then loaded with a Rhod-2 AM dye (1 µM) (K_d_ = 570 nM, λ_Ex_ = 552 nm and λ_Em_ = 581) (Invitrogen, CA, USA) for 45 minutes. After the dye loading, the cells were rinsed, incubated with either normal Hanks' or Ca^2+^-free Hanks' solution, and treated with the appropriate TiO_2_ concentrations. All calcium signaling experiments were carried out on a thermoregulated stage at 37°C mounted on a Nikon microscope (Nikon Eclipse TE2000-U, Tokyo, Japan). ChaGo-K1 cells were incubated with cadmium chloride (200 µM; Sigma-Aldrich, MO, USA) to block the membrane Ca^2+^ channels [34], followed by TiO_2_ NP stimulation. To investigate the interaction between TiO_2_ and membrane Ca^2+^ channels, nifedipine (10 µM; Sigma-Aldrich, MO, USA), an L-type Ca^2+^ channel blocker [27], was added to ChaGo-K1 cells prior to the exposure of TiO_2_. Antioxidant N-acetylcysteine (NAC, 250 µM; Sigma-Aldrich, MO, USA) was also added to ChaGo-K1 cells to study the involvement of reactive oxygen species (ROS) [27], [35], possibly generated as a result of TiO_2_ stimulation, and the activation of Ca^2+^ channels. Thapsigargin (100 nM; Sigma-Aldrich, MO, USA) [18] and ryanodine (100 µM; Sigma-Aldrich, MO, USA) were added separately to deplete the ER Ca^2+^ content and to inhibit the CICR mechanism [20], [21], correspondingly. These two blockers were utilized to investigate the contribution from the internal ER Ca^2+^ pool.

### 5. Calcein dye leakage measurements

ChaGo-K1 cells were seeded at the density of 2×10^5^ cells per well in a 24-well plate and cultured for 24 hrs. TiO_2_ NP prepared with calcein fluorescent dye (50 µM) (Invitrogen, CA, USA) in Hanks' solution was incubated with the cells for 5 minutes at 37°C. Calcein is a biological inert green-fluorescent molecule of a molecular mass of 623 Da and an estimated molecular radius of 0.6 nm [36]. TiO_2_ NP solution containing the calcein dye was then removed and cells were rinsed twice with PBS to remove possible remnants of calcein dye in the extracellular solution. The cells were subsequently stained with a fluorescent nucleus dye, hoechst (10 µM) (Sigma-Aldrich, MO, USA), for 5 mintues at 37°C and thoroughly rinsed again [33]. Fresh Hanks' solution was added into each well before taking fluorescent images of calcein and hoechst loaded cells with a Nikon fluorescence microscope. A percentage of calcein loaded cells against total number of cells, as indicated by hoechst fluorescence, was calculated for each of the TiO_2_ NP concentrations used in the experiment.

### 6. Mucin secretion and ELLA Preparation

The cells were seeded at 2×10^5^ cells per well in a 24-well plate and cultured for 24 hrs. ChaGo-K1 cells were then rinsed with PBS and treated with BAPTA-AM (Invitrogen, CA, USA), thapsigargin (Sigma-Aldrich, MO, USA) or ryanodine (Sigma-Aldrich, MO, USA) for at least 30 minutes. Afterward the cells were stimulated for 15 minutes with the corresponding TiO_2_ NP concentrations (0.75 mg/ml, 0.5 mg/ml, 0.25 mg/ml, and 0.1 mg/ml) or ionomycin (1 µM) (positive control) (Sigma-Aldrich, MO, USA), both prepared in PBS. The supernatant containing secreted mucin was collected and briefly centrifuged at 8,000 rpm to remove the residual TiO_2_ NPs. The supernatant was then incubated in a 96 well (Nunc MaxiSorp, VWR, CA, USA) plate overnight at 4°C. Afterward the 96-well plate was washed with PBST (PBS + 0.05% Tween-20) and then blocked with 1% BSA. The 96 well plate was washed again with PBST and incubated with lectin (Wheat germ agglutinin, WGA) (Sigma-Aldrich, MO, USA), conjugated to horseradish peroxidase (HRP; 5 µg/ml) (Sigma-Aldrich, MO, USA), at 37°C for 1 hr. The substrate, 3,3′,5,5′-Tetramethylbenzidine (TMB; Sigma-Aldrich, MO, USA), was added to each well at room temperature followed by H_2_SO_4_ (Sigma-Aldrich, MO, USA) in order to terminate the reaction. The optical density was measured at 450 nm [37].

### 7. Image Analysis

After staining the treated cells, image analysis was performed with an inverted Nikon Eclipse TE2000-U fluorescent microscope. Each photo was taken at a magnification of 200× and analyzed using SimplePCI (Compix Inc., Imaging Systems, Sewickle, PA, USA). The data shown is a representative of Ca^2+^ signals of more than 200 cells.

### 8. Statistical Analysis

The data was presented as means±SD. Each experiment was performed independently at least three times. Statistical significance was determined using a Student's t-test analysis with p values <0.05 (GraphPad Prism 4.0, GraphPad Software, Inc., San Diego, CA, USA).

## Results

### TiO_2_ NP characterization

**Figure 1:**
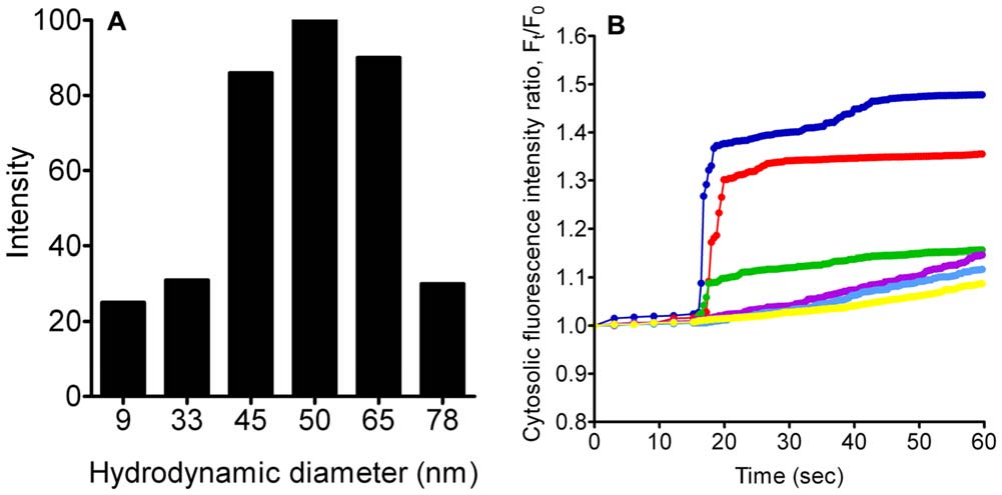
TiO_2_ NP characterization and resultant [Ca^2+^]_C_ changes after NP treatment. A) DLS assessment of TiO_2_ NPs in Hanks' solution showed a size distribution of ∼9 to 80 nm. B) Cells were treated with TiO_2_ NPs with concentrations of 0.05 mg/ml (yellow), 0.1 mg/ml (Light Blue), 0.25 mg/ml (Purple), 0.5 mg/ml (Green), 0.75 mg/ml (Red), and 1 mg/ml (Blue) in normal Hanks' solution. Each line represents the average fluorescence intensity of approximately 200 cells per well.

Dynamic laser scattering (DLS) was used to characterize the TiO_2_ NPs. The particle size distribution ranged from ∼9 to 80 nm due to minor aggregation or agglomeration while the predominant size is ∼50 nm (Fig. 1A).

### TiO_2_ NPs induce cytosolic Ca^2+^ concentration increase

To investigate whether TiO_2_ NPs could generate an increase in [Ca^2+^]_C_, ChaGo-K1 cells were loaded with Rhod-2 AM dye and exposed to 0.05–1 mg/ml of TiO_2_ NPs. The change in [Ca^2+^]_C_, as represented by the fluorescence intensity within ChaGo-K1 cells, was monitored for 60 seconds. Figure 1B shows that 1 mg/ml of TiO_2_ NPs induced an approximate 150% increase, while lower TiO_2_ concentrations (<0.1 mg/ml) caused a minor elevation (∼110%) in [Ca^2+^]_C_ when compared with untreated cells. The effect of TiO_2_ treatment on the [Ca^2+^]_C_ of ChaGo-K1 cells followed a concentration-dependent manner (Fig. 1B).

### Extracellular source for Ca^2+^ increase

**Figure 2:**
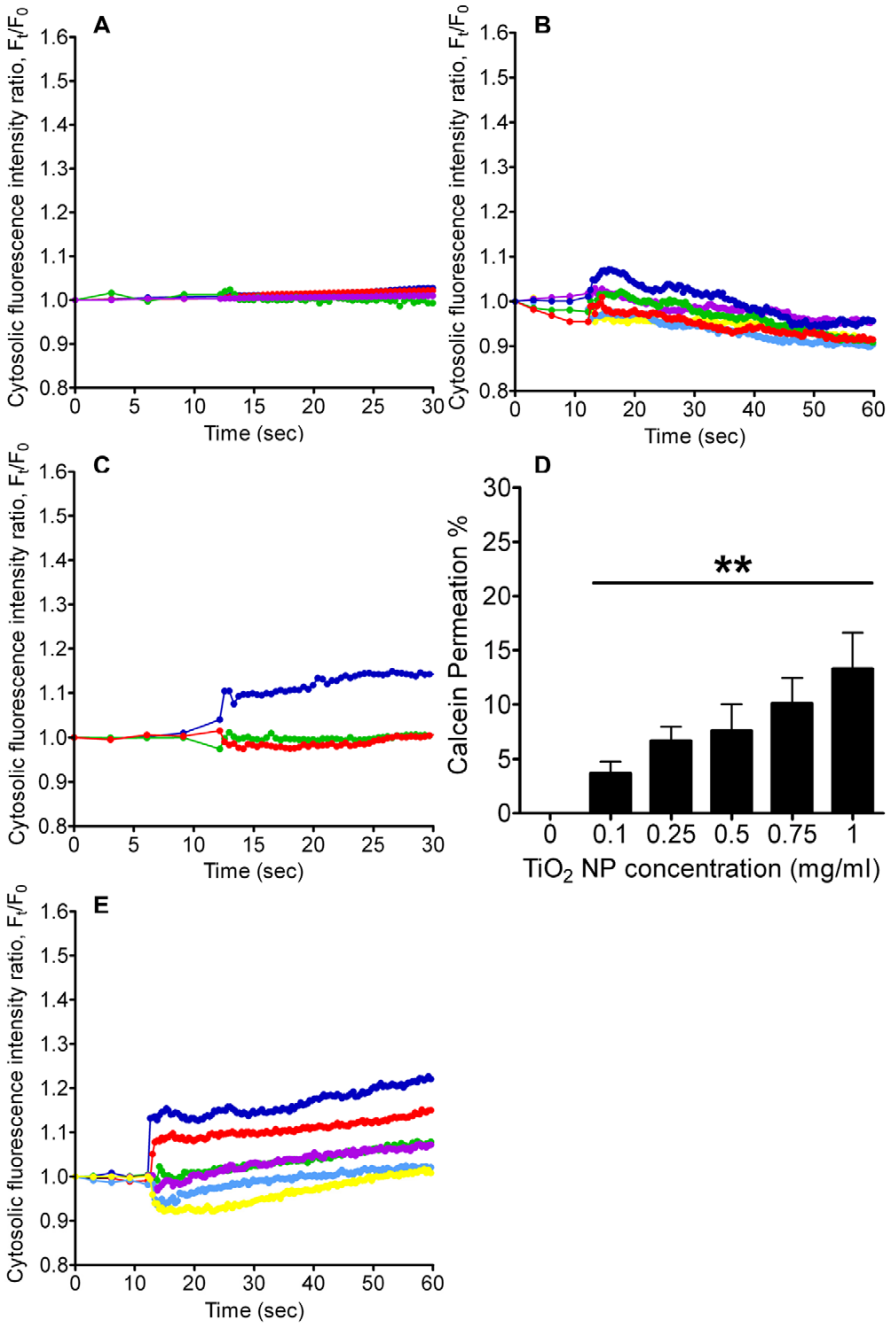
Measurement of the [Ca^2+^]_C_ and calcein dye leakage after TiO_2_ NP treatment. Cells were treated with TiO_2_ NPs with concentrations ranging from 0.05 mg/ml–1 mg/ml, in A) Ca^2+^-free Hanks' solution, B) in the presence of CdCl_2_ (200 µM), C) nifedipine (10 µM), D) calcein (50 µM) (n = 12, **P<0.005), and E) NAC (250 µM) (colors are as depicted in Figure 1B).

To determine the main source of elevated [Ca^2+^]_C_ upon stimulation, ChaGo-K1 cells were exposed to TiO_2_ NPs in Ca^2+^-free Hanks' solution. EGTA (2 mM) was added in Hanks' solution to chelate possible traces of Ca^2+^. TiO_2_ (0.05 mg/ml–1 mg/ml) treatment under Ca^2+^-free conditions failed to instigate a significant increase in [Ca^2+^]_C_ (Fig. 2A). Our data suggests that the extracellular Ca^2+^ pool is the primary source of the observed cytosolic Ca^2+^ increase. We then tested whether TiO_2_ NPs can induce a Ca^2+^ influx via membrane channels. Blocking the channels with CdCl_2_ (200 µM) significantly inhibited an increase in [Ca^2+^]_C_ (Fig. 2B). Co-treatment of cells with TiO_2_ NPs and nifedipine greatly blocked the NP-induced [Ca^2+^]_C_ increase (Fig. 2C). However, the incomplete blockage of extracellular Ca^2+^ influx via channels postulates additional Ca^2+^ leakage through perturbed cell membranes. To confirm whether TiO_2_ can instigate membrane disruption, thereby permitting unspecific extracellular Ca^2+^ entry, cytosolic leakage was assessed using the fluorescent calcein dye. It was found that the dye permeation ratio increased from approximately 4 to 13% with elevated TiO_2_ concentrations ranging from 0.1 to 1 mg/ml (Fig. 2D).

### Oxidative stress induced Ca^2+^ influx

To demonstrate that TiO_2_-evoked [Ca^2+^]_C_ increase can be associated with oxidative stress, cells were pretreated with an anti-oxidant, N-acetylcysteine (NAC) [27]. Pre-treatment with NAC was able to partially attenuate the increase in cytosolic Ca^2+^ level triggered by 1 mg/ml and 0.75 mg/ml TiO_2_ exposure (Fig. 2E). These results support the idea that oxidative stress, induced by TiO_2_ NPs, contributes to the observed [Ca^2+^]_C_ increase and promote Ca^2+^-dependent mucin secretion.

### The ER as an intracellular source of Ca^2+^

**Figure 3:**
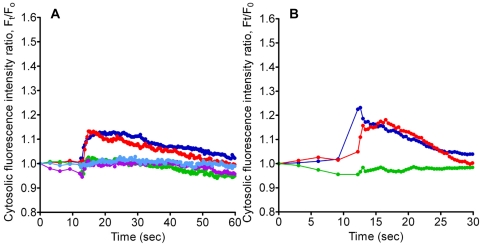
Measurement of [Ca^2+^]_C_ after stimulation by TiO_2_ NPs. Cells were treated with TiO_2_ NPs with concentrations ranging from 0.1 mg/ml–1 mg/ml, in the presence of A) thapsigargin (100 nM), and B) ryanodine (100 µM) (colors used are consistent with Figure 1).

In order to determine the involvement of ER Ca^2+^ pool, it was depleted by pre-incubating the cells with thapsigargin. Pre-treatment with thapsigargin impeded TiO_2_ NPs from triggering a sustained increase in the cytosolic Ca^2+^ level (Fig. 3A). We then investigated the role of the CICR mechanism by blocking RYRs (ryanodine receptors) [20]. Our results revealed that CICR was largely inhibited by ryanodine (a blocker for RYR associated with the CICR response) resulting in a significantly diminished [Ca^2+^]_C_ increase induced by NPs (Fig. 3B).

### Ca^2+^-dependency of TiO_2_-induced mucin secretion

**Figure 4:**
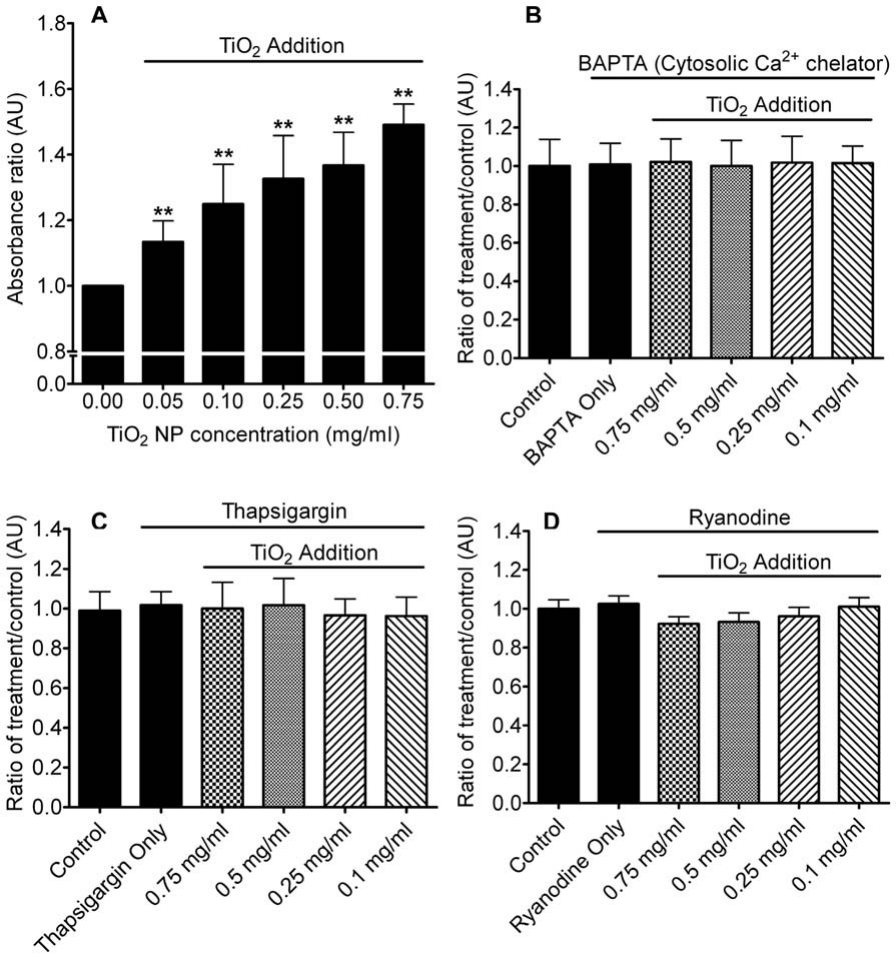
Measurement of mucin secretion triggered by TiO_2_ NPs. Cells were treated with TiO_2_ NP concentrations ranging from 0.05 mg/ml–0.75 mg/ml. Figure 4A) shows the relative quantification of mucin secreted after TiO_2_ stimulation under normal conditions (n≥7, **P<0.005), 4B) in the presence of BAPTA-AM (50 µM) (n≥9), 4C) with pre-treatment of thapsigargin (100 nM) (n≥8), 4D), and with ryanodine (100 µM) (n≥5).

**Figure 5:**
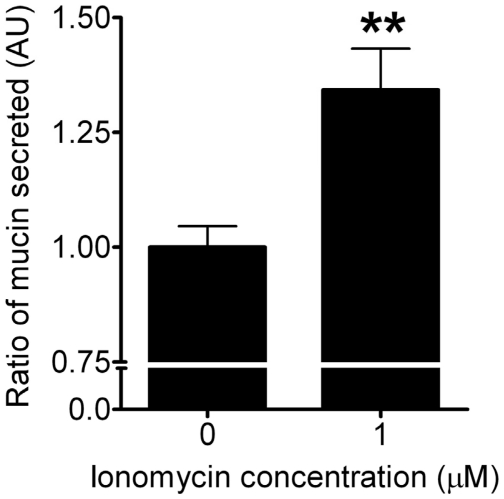
Mucin secretion in response to ionomycin application (positive control, n≥3). Concentration of ionomycin used was 1 µM.

Enzyme-linked lectin assay (ELLA) was used to assess the amount of mucin secreted from ChaGo-K1 cells when stimulated with TiO_2_ NPs. When compared to the control, TiO_2_ NPs increased mucin secretion by 113%, 125%, 133%, 137% and 150% at 0.05, 0.1, 0.25, 0.5 and 0.75 mg/ml, respectively (Fig. 4A). Chelating the intracellular Ca^2+^ with BAPTA-AM yielded a significant reduction in mucin secretion (Fig 4B). Addition of thapsigargin (Fig. 4C) or ryanodine (Fig. 4D) also resulted in diminished mucin secretion induced by TiO_2_ NPs. Our data indicates that TiO_2_-induced mucin secretion is dependent on the [Ca^2+^]_C_, attributed to both external and internal Ca^2+^ pools (Fig. 4A–D). Ionomycin (a Ca^2+^ ionophore) was used to elicit mucin secretion as a positive control (Fig. 5).

## Discussion

Recently, an increasing number of reports have shown that airborne particulate pollution found in both the ambient and working environments, particularly TiO_2_ NPs, can exacerbate airway diseases [1], [2], [3], [4], [5], [6], [10], [11], [38]. Aggravated clinical manifestations of COPD, CF and asthma may include intensified symptoms of mucociliary transport impairment and mucus hypersecretion [15], [39]. The resultant accumulation of thick obstructive mucus usually occupies airway lumen, thereby limiting airflow and leading to morbidity [15], [39]. Despite documentations of TiO_2_-induced cellular nanotoxicity effects, pulmonary inflammatory responses and emphysema-like pathology [12], whether TiO_2_ NPs can directly trigger mucin secretion has not been resolved. In this study, we demonstrate that TiO_2_ NPs can stimulate mucin secretion from bronchial epithelial ChaGo-K1 cells via a Ca^2+^- dependent pathway.

Our study showed that TiO_2_ NPs can induce mucin secretion that increases as a function of TiO_2_ NP concentration (Fig. 4A). The TiO_2_ concentration range used in our study is consistent with previous reports representing the concentration found in ambience and nanotechnology industries [30], [40], [41], [42]. While NP exposure has been long associated with increasing mucin synthesis due to goblet cell hyperplasia [13], our study indicates that TiO_2_ NPs can directly trigger mucin secretion in the airway.

It has been well established that intracellular Ca^2+^ plays a vital role in stimulus-secretion coupling [43]. Previous reports have documented that an elevated [Ca^2+^]_C_ precedes mucin granule exocytosis [17]. NP exposure has been shown to trigger an intracellular Ca^2+^ increase in various cells; therefore, we examined the cellular Ca^2+^ signaling pathway involved during TiO_2_ stimulation [25], [28], [44]. At TiO_2_ concentrations of 0.5, 0.75, and 1 mg/ml, there was a sustained elevation in [Ca^2+^]_C_. At lower doses (0.05, 0.1 and 0.25 mg/ml), the [Ca^2+^]_C_ increased gradually within the 1^st^ minute (Fig. 1B). Our data demonstrated that TiO_2_ NPs induced a concentration dependent increase in [Ca^2+^]_C_, which is consistent with results from the mucin secretion measurements (Fig. 4A).

The stimulus-induced intracellular Ca^2+^ signal can be evoked by the entry of Ca^2+^ through voltage-gated Ca^2+^ channels, or by the release of Ca^2+^ from intracellular Ca^2+^ stores [43], [45], [46]. Previous researches have suggested that extracellular Ca^2+^ influx plays an important role in the elevated [Ca^2+^]_C_ during NP stimulation [25], [27], [28], [47]. Data from experiments performed in Ca^2+^-free Hanks' solution confirmed that [Ca^2+^]_C_ failed to increase when treated with TiO_2_ NPs (Fig. 2A). To characterize the nature of the Ca^2+^ influx induced by TiO_2_ NPs, we first evaluated the effect of cadmium chloride (CdCl_2_), a general Ca^2+^ channel blocker [34], [48]. Figure 2B shows that the [Ca^2+^]_C_ remained low and relatively unchanged with CdCl_2_. Secondly, nifedipine, a widely used L-type Ca^2+^ channel blocker, markedly diminished the increase in [Ca^2+^]_C_ (Fig. 2C). The effect of nifedipine implies that TiO_2_ NPs can activate L-type voltage gated Ca^2+^ channels, allowing extracellular Ca^2+^ influx into the cytosol. This observation is consistent with previous reports showing that ultrafine carbon black and ZnO NP-induced [Ca^2+^]_C_ elevation can also be attenuated by nifedipine [27], [28]. In addition, several reports have suggested that oxidative stress induced by NPs can exert an impact on the intracellular Ca^2+^ signaling pathway and that the activity of Ca^2+^ channels may be altered by ROS [27], [28], [44]. Results from Figure 2E showed that NAC significantly reduced the rising [Ca^2+^]_C_ generated by TiO2 NPs. Huang et al, has also demonstrated that NAC can attenuate the intracellular Ca^2+^ level when challenged with ZnO NPs [27]. Our results support the idea that NAC and other antioxidants may be effective in reducing NP-instigated mucin hypersecretion. NPs such as TiO_2_ can damage cell membrane integrity by possible lipid peroxidation [27], [31], thereby creating pores on the lipid bilayer [49] that may allow the transient influx of extracellular Ca^2+^. Our data further demonstrated that co-adminstration of TiO_2_ NPs and fluorescent calcein dye lead to intracellular leakage and the permeation efficiency increased in a TiO_2_ concentration dependent manner (Fig. 2D). Calcein has also been previously utilized to evaluate the efficacy of peptides in causing membrane perturbation [50]. Our result suggests that the possible membrane perturbation/transient pore formation induced by TiO_2_ NPs allows an extracellular Ca^2+^ influx and may account for the portion of Ca^2+^ that can not be completely abolished by blocking L-type Ca^2+^ channels with nifedipine.

Increasing the [Ca^2+^]_C_ of human goblet cells has been shown to trigger degranulation [17]. We used BAPTA (cytosolic Ca^2+^ chelator) to test whether the increase in Ca^2+^ induced by TiO_2_ NPs could stimulate mucin exocytosis. It is evident that BAPTA significantly inhibited mucin exocytosis (Fig. 4B), indicating that TiO_2_ NPs can elicit a [Ca^2+^]_C_ increase, thereby leading to mucin secretion.

Besides the external Ca^2+^ source (Hanks' solution), the ER is one of the major internal Ca^2+^ stores. Figures 3A and 4C revealed that when the ER Ca^2+^ had been depleted by pretreatment with thapsigargin, the TiO_2_ NP-induced [Ca^2+^]_C_ failed to increase significantly, and the subsequent mucin secretion was abolished. Our data indicates that the ER plays a critical role in relaying TiO_2_-induced Ca^2+^ signaling. CICR is a positive feedback mechanism where the ER amplifies a small increase in [Ca^2+^]_C_, (e.g. due to voltage-gated Ca^2+^ influx [22]), with the activation of RYRs that will lead to the release of more Ca^2+^ from the ER [19], [20]. Previous studies have shown that through activation of RYRs with Ca^2+^, CICR can generate an overall increase in [Ca^2+^]_C_ [20], [21], [22]. Our data showed that ryanodine inhibited a continual rise in [Ca^2+^]_C_ when applying TiO_2_ NPs (Fig. 3B). Therefore, it is indicative that the TiO_2_-instigated increase in [Ca^2+^]_C_ was also CICR dependent. The effect of ryanodine was further demonstrated by the lack of mucin secretion under TiO_2_ NP stimulation (Fig. 4D).

In summary, our study indicates that cellular exposure to TiO_2_ NPs can activate membrane L-type Ca^2+^ channels, induce ROS production and possibly disrupt the cellular membrane. Influx of extracellular Ca^2+^ into the cytoplasm raises [Ca^2+^]_C_, which in turn can trigger ryanodine receptors on the ER to release ER resident Ca^2+^ via the CICR mechanism. A sufficient increase in the cytosolic Ca^2+^ level results in subsequent mucin secretion. More importantly, our results provide a direct link between airborne particulate matters and the pathogenesis of chronic airway diseases involving mucus hypersecretion and airway obstruction. In addition, we demonstrate that once thought inert and harmless TiO_2_ NPs can indeed interfere with intracellular Ca^2+^ signaling, possibly leading to pathological states.
